# Correlation between Resistin, Tuberculosis and Khat Addiction: A Study from South Western Province of Saudi Arabia

**DOI:** 10.1371/journal.pone.0140245

**Published:** 2015-10-08

**Authors:** Ayesha Alvi, Nuzhath Fatima, Ahmed Ali Jerah, Mohammed Rizwan, Yahya Hasan Hobani, Rashad Al Sunosi, Manal Mohamed El Hassan Taha, Eldaw Mohamed Habiballah, Pradeep Kumar Agarwal, Siddig Ibrahim Abdulwahab

**Affiliations:** 1 Department of Genetics and Molecular Biology, Faculty of Applied Medical Sciences, Jazan University, Jazan, KSA; 2 Department of Microbiology, Faculty of Applied Medical Sciences, Jazan University, Jazan, KSA; 3 Department of Biochemistry, Faculty of Applied Medical Sciences, Jazan University, Jazan, KSA; 4 Department of Biochemistry, College of Nursing, Jazan University, Jazan, KSA; 5 Substance Abuse Research Centre, Jazan University, Jazan KSA; 6 Department of Biomedical Research unit, Medical Research Centre, Jazan University, Jazan, KSA; 7 Chest Hospital, Abu Areesh, Jazan KSA; Indian Institute of Technology Delhi, INDIA

## Abstract

Tuberculosis(TB) is a disease of global significance, which accounts for a death in every 15 seconds. Recent studies shows TB is rising in certain parts of the world, and Saudi Arabia is one of them. Several factor contribute in predisposing the subjects for infection including but not limited to addiction to various compounds which have immune modulation properties, such as amphetamines and Heroin etc. Khat a plant whose leaves are chewed for its euphoric effect in east Africa and Arabian Peninsula including Saudi Arabia, is considered as mildly addictive, and its principle compound, Cathinone shares structural and functional similarity with amphetamine a known immunomodulator. Tuberculosis being a disease of immune modulation has a varied spectrum of complex interplay of proinflammatory molecules, resistin is one of them. In the present study, we try to explore the trinity of khat addiction, serum resistin level and tuberculosis by correlating the serum resistin level in non khat addicted healthy subjects, khat addicted healthy subjects, and in patients, both khat addicted and non khat addicted, with active tuberculosis. We observed significantly higher resistin level among the apparently healthy khat addicted subjects as compared to non addicted healthy controls. Thereafter, when we compare the resistin levels between khat addicted and non khat addicted TB patients we did not found significant difference between the two groups. However bacillary load was observe to be significantly higher among the khat addicted TB patient as compare to non addicted one. Validation of above results in animal model revealed dose dependant increase in bacillary growth in the Wistar rats treated with khat. Taken together these results suggest the role of khat in immune modulation albeit in the limited frame of resistin level.

## Introduction

Tuberculosis (TB) is a chronic inflammatory disease caused by *Mycobacterium tuberculosis* (Mtb). Global estimates shows 9 million people are suffering from this disease. TB is ranked as second most leading cause of death by infectious agent after HIV specially in the developing countries [1]. In recent years, Saudi Arabia is witnessing a steep rise in the number of tuberculosis cases, apparently several factors have been argued to regulate the dissemination dynamics of disease such as a recent boom in economic activity in the KSA which facilitated high influx of workers from different endemic regions like Asia and Africa, annual visit of more than three million pilgrims to the holy cities and increased population densities [2–4] etc. This analogy is further testified by National TB surveillance data by Ministry of Health, KSA, which revealed high incidence rate particularly in the Central region, Makkah and in the Southern western province Jazan [4]. The data indicated that despite nationwide coverage of DOTS programme TB prevalence did not fall as expected notably in the Jazan region. The higher incidence rate (18/100,000) recorded in region calls for monitoring additional factors to tackle the pestilence. Jazan being the border area houses immigrants from countries like Yemen, Somalia and Africa where khat is chewed culturally and traditionally for its euphorigenic effect. In principle this promote its availability and its usage by Saudi nationals as well [5]. Khat is an evergreen shrub, when fresh leaves and shoots are chewed; its active constituents like cathinone dissolve in saliva and readily absorbed in the blood to elicit its ill effect on the health [6, 7].Cathinone shares structural and functional similarity with amphetamine; which is a known immunosuppressive addictive compound[8]. It has been shown to have immune modulatory effect on macrophages. Suppression of immune competence among the substance abuser usually pave favourable niche for opportunistic infections like HIV and *Mycobacterium* [9]. Given to this structural and functional similarity with amphetamine we hypothesized cathinone or active components of khat possibly shares cross talks with immune system to egg on infectious diseases or predisposes its addicts to opportunistic infection by playing with proinflammatory molecules such as resistin. Therefore khat chewing possibly corroborate with the fact that Tb infection is on the rise, despite effective anti TB programmes in Jazan, KSA [4, 10]. Taken together, we consider analyzing the association between khat addiction, tuberculosis and resistin levels.

Resistin, is a cysteine rich unique proinflammatory signalling molecule predominantly secreted in humans by macrophages[11] Various studies positively demonstrated it role in chronic inflammatory diseases such as atherosclerosis, endotoxemia, asthma, coronary artery disease and arthritis etc [12–16]. Nonetheless, the ability of resistin to induce a Th1 response[17, 18] also explicates its role in infectious diseases via innate immune mechanisms. Moreover detection of systemic hyper resistinemia in severe, sepsis or septic shock also suggests a plausible role of resistin in infectious disease[19, 20] Given to these, the study was design to determine firstly the circulating serum resistin level among the healthy subjects not addicted to khat and healthy subjects addicted to khat, to ascertain whether khat addiction does have an effect on serum resistin levels, followed by assessing their levels in khat addicted and non khat addicted tuberculosis patients. Secondly, we evaluate the invivo effect of khat in wistar rat using *Mycobacterium*. *smegmatis* bacilli as non pathogenic model mimicking the *Mycobacterium*. *tuberculosis* infection to assess whether khat facilitates the growth of mycobacterium in lungs This study will be first of its kind to assess a correlation between resistin level, among khat addicted and non addicted subjects and tuberculosis patients with and without khat addiction.

## Material and Methods

### Study Design

Patient’s samples were collected from Chest hospital, Abu Arish, Jazan, KSA. Our study group consist of four different categories 1) non khat addicted subjects (n = 72) freshly diagnosed as having active- pulmonary TB by clinical symptoms (fever, cough and positive chest x-ray) and positive sputum smear test. 2) khat addicted subjects with active tuberculosis (n = 49) 3) khat addicted healthy control (n = 48) 4) non khat addicted healthy control (n = 50). Upon enrolment patients were administered with standard anti- tuberculosis drug under DOTS programme and were monitored for a period of six months for follow up therapy. During follow up visits demographic information were documented through a structured questionnaire setup. Patients with myocardial infarction or other respiratory disorders, diabetes, renal failure, hypertension, pregnancy or jaundice were excluded from the study. Their mean age was 38 yrs (range 19–59 years). Finally 50 apparently healthy subjects with no respiratory symptoms were also recruited in the study as case controls (21 males and 29 females) with a mean age of 28 yrs (range 20–50 yrs). None of the subjects enrolled in the study had any clinical evidence of co-infection with human immunodeficiency virus (HIV). Informed written consent was obtained from all the subjects prior to their enrolment in the study according to the institutional ethical guidelines. Study was approved by ethical committee of Faculty of Medicine, Jazan University

### Follow-up study design and specimen collection

During therapy and follow up visit patients were clinically assessed for the evidence of active tuberculosis and sputum specimens were sent for staining. 2ml venous blood samples were collected at first visit (0 month) and at every two months interval (2, 4 and 6). Serum was separated and stored at -70°C until assayed.

### Collection and analysis of Sputum samples collected from tuberculosis patients

Following clinical diagnosis of tuberculosis, sputum samples collected from the patients were first decontaminated according to the standard sodium hydroxide–N-acetyl-L-cysteine (NALC) method[21].The samples were concentrated by centrifugation at 4,000Xg for 10 minutes and the sediments smears were made, and stained by Ziehl-Neelsen, examined for AFB. The severity of tuberculosis was graded according to the guidelines of Revised National Tuberculosis Control Program (RNTCP) and the patients were categorised into three groups (1+, 2+ +, 3+ + +) depending upon the severity of infection.

### ELISA for estimating serum resistin levels

Serum resistin levels were measured using commercially available enzyme-linked immunosorbent kit (Assaypro, USA). The assay was essentially performed as per the instructions of manufacturer following all the recommended quality control procedures. All the sera (diluted 1:20) were tested in duplicate to ensure greater inter-assay precision of the results. The concentration of resistin was calculated by interpolation of regression curve formula recommended by the manufacturer. The detection limit of the kit was 100pg/ml resistin.

### Preparation of crude extract from Catha edulis (Khat)

Relatively fresh leaves of Catha edulis (Khat) were obtained from Substance Abuse Research Centre, Jazan University, Saudi Arabia. Khat leaves were washed in sterile water and air dried at room temperature. The leaves were weighed and soaked in 100 ml of PBS in a water bath at 25°C for 24 h with continuous shaking using Cole Parmer Orbital Shaker at 40 g. Following this, leaves were homogenized and the extract was filtered using Whattman No1filter paper and concentrated at 40°C under reduced pressure. The crude extract was finally diluted to obtain a final dose concentration of 50, 100, 200 mg/ml.

#### Bacterial strain preparation

*Mycobacterium smegmatis* strain was cultured in Middlebrook broth to mid log phase to obtain an OD_600_ of 1.2 corresponding to approximately 1x10^8^ CFU/ml.

### Determining In vivo response to khat

To mimic the effects of addiction of khat in vivo, we conducted a randomized animal experiment wherein adult male Wistar rats were divided into 4 groups of five animals each. Rats, approximately 6–8 weeks old at the start of the experiments, were fed with a standard sterilised laboratory chow and given water ad libidum. They were placed in ventilated cages (3 animals per cage) under controlled room temperature of 25°C ± 2°C with a 12:12 hour light: dark cycle (lights on at 6 a.m). Following one week of acclimatization, three group animals were infected endotracheally with the bacillary suspension of *Mycobacterium smegmatis* for a period of four weeks to establish course of infection[22]. Briefly, a plastic needle is passed through the mouth and trachea of the anaesthetized animal as described earlier[23]. Later, the first group of animals were fed with a daily single dose of different concentrations of khat extract (50, 100, 200 mg/Kg) for one week [24]. While the second group, was orally administered with a daily single dose of anti- TB drug (Rifampacin 30mg/kg) for a period of one week [22] Control animals infected with bacilli were given saline only. At the end of the treatment period animals were sacrificed by decapitation and their lungs were aseptically excise from the pulmonary cavity for CFU estimation. The study was conducted in accordance with internationally accepted rules for laboratory animal use and care (NIH# 85–23 revised 1985). Ethical approval was obtained from the ethical committee of Animal care, Faculty of Medicine, Jazan University.

### Enumerating Mycobacterial count in Lungs

Lungs were washed in PBS and homogenized using a tissue homogenizer (WiseTis, Germany) homogenates were serially diluted in sterile PBS and plated on Middlebrook 7H11 agar plates (Difco Laboratories) and incubated at 37°C to determine the bacterial count.

### Statistical analysis

Statistical analyses were performed for different groups using graph pad software (*www*.***graphpad***.*com/quickcalcs/* *)*. Mean titres were compared by Student's *t* test and the values were expressed as means ± standard deviation. For all the analyses *P* ≤ 0.05 were considered as statistically significant. To determine the cut off values, mean OD+2SD of healthy individual was calculated and the sera with values above the cut off line was considered as risk factor for disease activity.

## Result

### Association of Serum resistin levels with khat addiction

**Fig 1:**
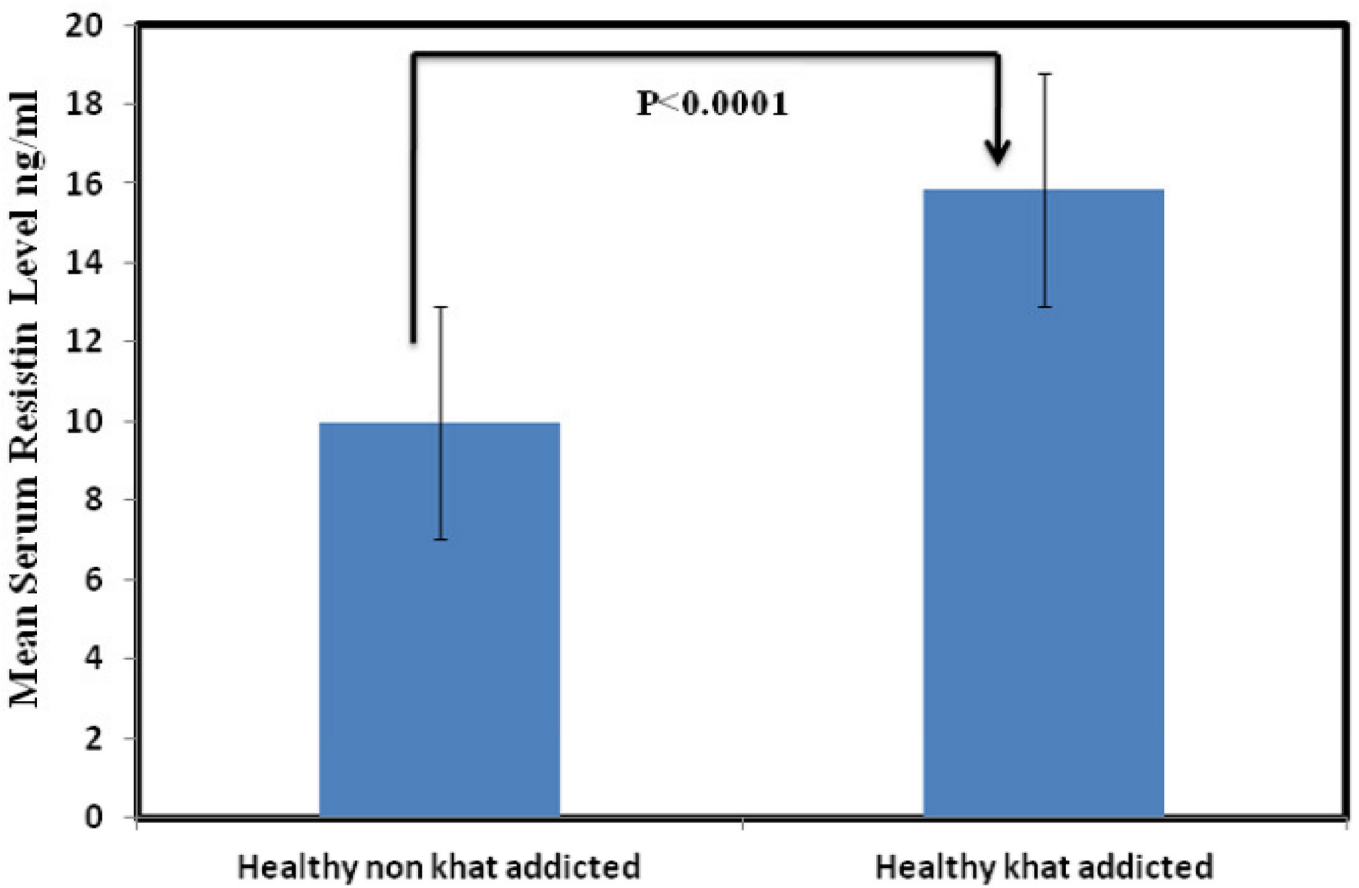
Comparison of mean resistin levels between Non khat addicted and khat addicted subjects. Serum resistin is significantly up regulated among the khat addicted healthy subjects.

To identify the effect of khat addiction on resistin a proinflammatory cytokine, we statistically analyzed and compare the levels of resistin between khat addicted apparently healthy individuals and non khat addicted healthy subjects. The data demonstrated that mean value of serum resistin levels (Mean ± SD) in khat addicted healthy subjects is 15.83± 3.17 ng/ml as compared to 9.97± 1.79 ng/ml in non khat addicted healthy subjects “Fig 1”. This observation partly validate that indeed secretion of resistin level among the healthy khat addicted individuals is high and its statistically significant (P< 0.0001) however further investigations are needed to understand the mechanistic aspects of khat in stimulating the production of resistin from macrophages.

### Resistin levels between khat addicted tuberculosis and non khat addicted tuberculosis

**Fig 2:**
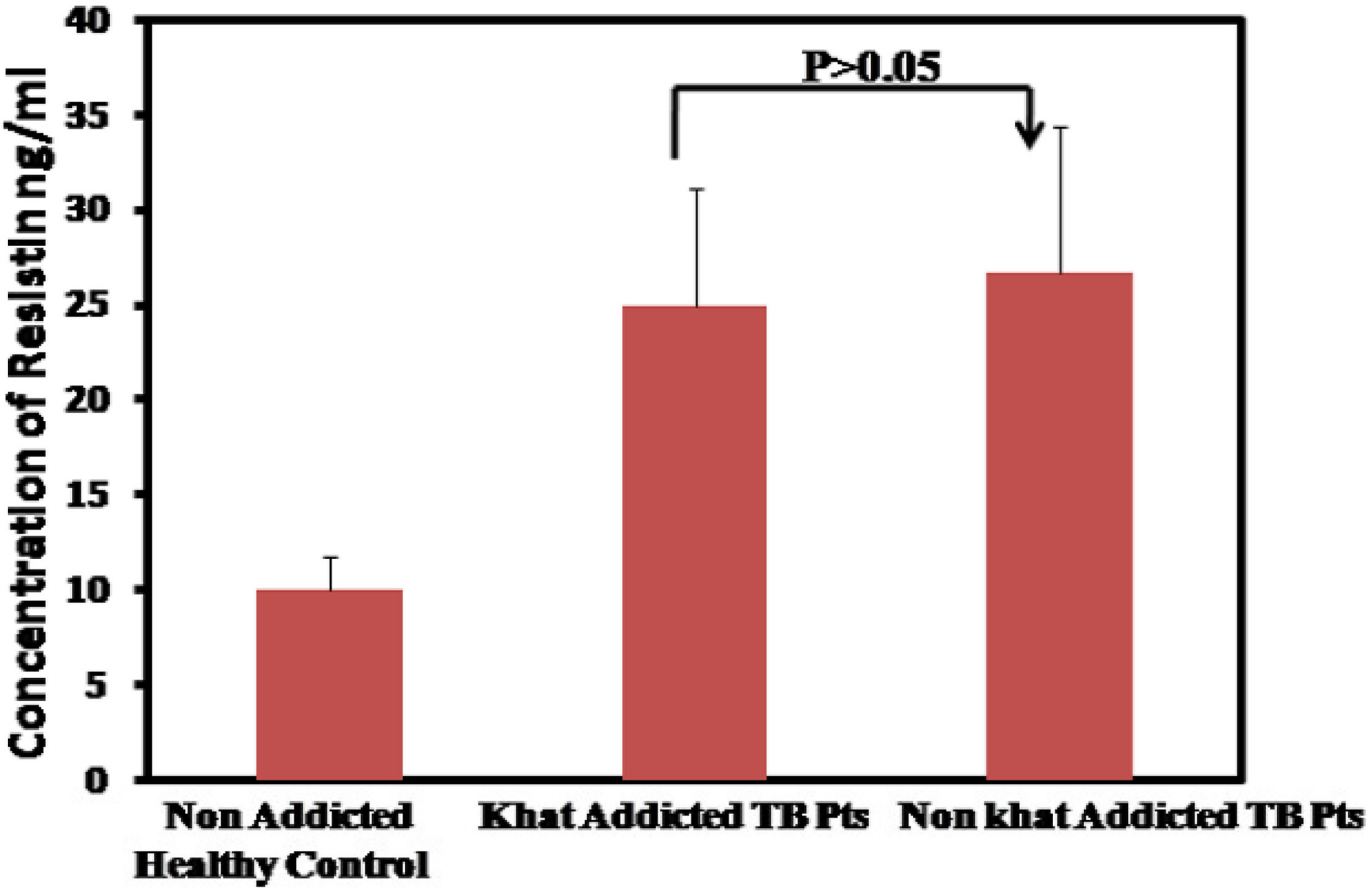
Elevated mean serum resistin level between khat addicted and non addicted Tuberculosis patients as compared to non addicted healthy controls.

To assess whether increased resistin levels among the khat abuser has any effect on severity of tuberculosis, we examined the resistin levels between khat addicted and non khat addicted tuberculosis patients. Surprisingly no statistically significant difference was observed between the two groups. Indicating that once seemingly up regulated in khat addicted subjects, resistin levels in khat addicted and non addicted subjects may solely be due to Tb infection. The mean resistin levels estimated in khat addicted TB patients is 26.69±7.67 ng/ml. Resistin did not correlate with age or sex in either patients or healthy control “Fig 2”.

### Serum resistin levels between pulmonary TB patients and healthy case control

**Fig 3:**
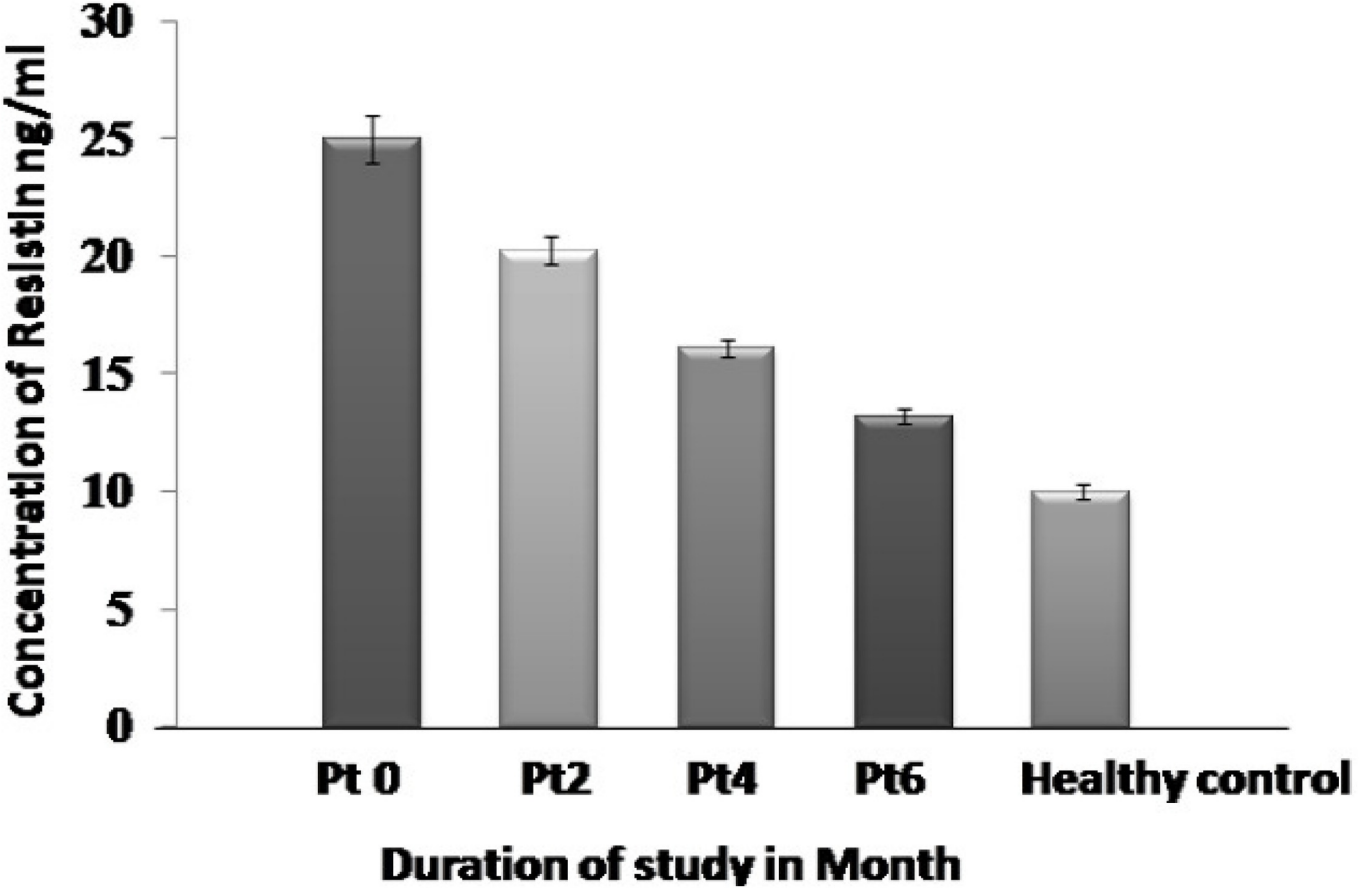
Correlation of Serum resistin levels between different groups of *Mtb* infected patients. Resistin decreases significantly during anti-TB therapy. Cut off value (13.0ng/ml) was calculated as the mean resistin values obtained in healthy control plus 2 standard deviations of the mean.

The mean serum circulating resistin levels are remarkably higher in TB patients (P0) as compared to apparently healthy non khat addicted subjects (24.98 ± 5.83 ng/ml vs 9.97± 1.79 ng/ml) and this difference is observed to be statistically very significant (P< 0.0001). Further, we measured circulating resistin levels in patient’s sera collected at different time points (0, 2, 4 and 6 month) during anti-TB therapy (ATT). It is interesting to note that there is a clear and significant decrease in the mean resistin levels following initiation of treatment. The mean titre values detected at 0 month is 24.98 ± 5.83 ng/ml as compared to 13.15 ± 2.51ng/ml at 6 months “Fig 3”.

### Relation between serum resistin levels and bacterial load

**Fig 4:**
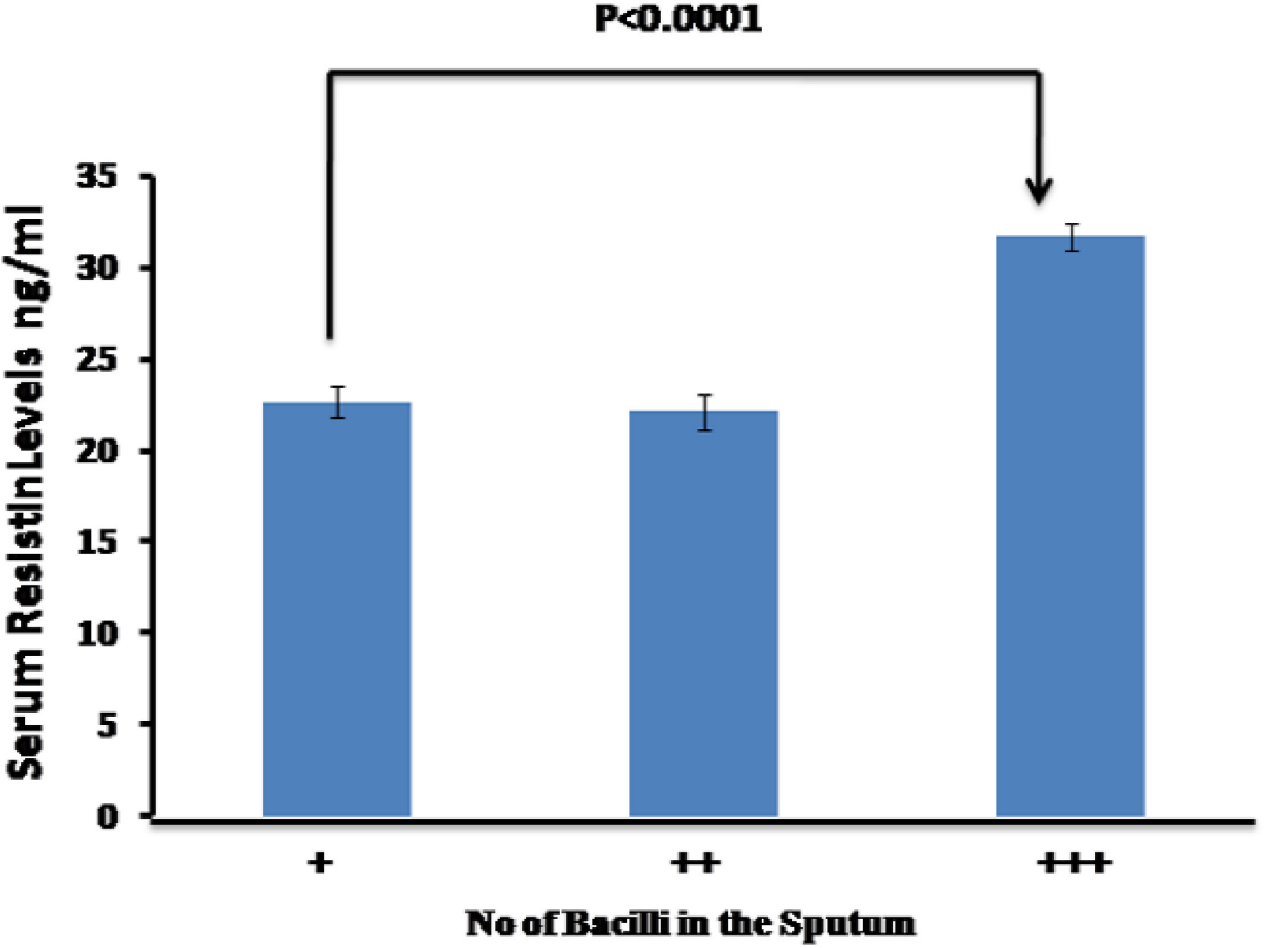
Mean serum resistin levels increases with bacterial load especially in the patients with chronic infection. Analysis of variance shows significant association between resistin and bacterial load P ˂0.05.

To evaluate whether resistin levels has any correlation with the severity of infection, we categorised the subjects into three groups before starting the ATT. Group one bacterial load is graded as 1+, group two 2 ++ and group three is 3 +++. The mean resistin values for group one is 22.65ng/ml (29 patients), group two 22.10 ng/ml (23 patients) and group 3 is 31.67 ng/ml (20 patients). Although no significant correlation was noted between the mean resistin values of group one and group two but statistically significant correlation was observed between group one and group three (P< 0.0001) “Fig 4”. Further, analysis of variance (One way ANOVA) of the above data gave F value of 32.355 which appears to be statistically insignificant (P>0.05). Contrastingly, resistin levels significantly correlate with bacterial load among the khat addicted tuberculosis patients (P<0.0006). ANOVA of bacterial load with resistin shows an F value of 13.56.

### Khat favours the growth of *M*. *Smegmatis*

To ascertain the effect of Khat extract on *Mycobacterium*. *smegmatis* in Wistar rats, we enumerate the bacillary growth following treatment with khat extract for a period of one week. Interestingly, a dose dependant increase in *Mycobacterium*. *smegmatis* growth was observed in contrast to anti Tb drug, rifampacin. Rifampacin shows good anti bactericidal activity as evident from reduction in the CFU 2.85X10^7.^ The effect was reproducible across two independent experiments “Table 1”.

**Table 1:** Invivo effect of Khat treatment on *M*. *smegmatis* growth.

S.No	Animal Group	CFU Counted Lung	Khat Dose mg/Kg		
			50	100	200
1.	Control (Only *M*. *Smegmatis*)	4.85X10^9^	-	—	
2.	*M*. *smegmatis*+ Khat	4.10X10^9^	5.2X10^9^	6.8X10^9^	16X10^9^
3.	*M*. *smegmatis* + Rif(30mg/Kg)	2.85X10^7^	-	-	-
4.	Control (Saline)	-	-	-	-

Results represented in the table are mean values calculated from two independent experiments.

## Discussion

Saudi Arabia is among one of the global hotspot for the growth and spread of tuberculosis. Its rather intriguing that despite high per capita income, relatively improved hygienic practices and a reasonable nutritional status and considerable investment in health care services and nationwide implementation of (NTP), TB trend is still on rise and facing several challenges. Therefore, there is a serious need to investigate the underlying cachexia to limit the dissemination of disease in the Kingdom especially in high risk regions like Makkah or Jazan. Khat addiction which is rampant in Jazan prompted us to look in to its role in immune modulation as its principle component cathinone shares structural and functional similarity with amphetamine a known immune modulator. We evaluated the levels of resistin in khat addicted and non khat addicted healthy subjects. The results of our study reveal significantly higher resistin levels among the khat abusing subjects as compared to unrelated healthy control(P>0.0001) “Fig 1”. This elevated resistin level reflects the possible effect of active compounds present in the khat in stimulating the production of resistin from macrophages. Given the understanding that resistin is a proinflammatory molecule hence elevated among the TB patients[25, 26], we thereafter, compared the serum levels of resistin between non khat addicted TB patients and khat addicted TB patients to validate the above stated analogy, interestingly, that although the difference in serum resistin levels between the two group did not reach statistical significance (P>0.05) but relatively higher serum resistin levels were detected among the khat addicted tuberculosis patients “Fig 2”. This is suggestive of the notion that although the levels of resistin among khat addicted and non khat addicted individuals does vary, considerably, once triggered apparently by khat chewing resistin levels among khat addicted and non khat addicted Tb patient remains same. Further investigations are needed to understand whether high resistin levels among apparently healthy khat addicted subjects predisposes them for opportunistic infection if yes by which way? Juxtaposing with the above results, bacillary load is recorded predominantly higher among the later group as compared to non khat addicted TB patients “Fig 4” This may be due to the fact that higher bacillary load triggers the production of resistin in an effort to impaired the release of reactive oxygen species important for controlling the growth of the bacterium [19, 27]. This even holds true given that resistin is a cysteine rich molecule and has an anti-oxidant effect [28–30]. We further investigated the effect of khat on *Mycobacterium*. *smegmatis* growth to assess whether khat promotes or facilitates its growth the results indicated that post khat treatment intracellular mycobacterium growth was strikingly increased as compared to control animals infected with *Mycobacterium*. *smegmatis* alone “Table 1”. Taken together, our findings point towards the likely role of khat in modulating the immune response, in this case *Mycobacterium smegmatis* possibly by suppressing the innate defense mechanism[19]. Additionally, these finding support the earlier studies which suggest the enhanced susceptibility of drug abuser to opportunistic infection[31].

To sum up it would be valuable to further expand this study to understand the mechanistic details of resistin up regulation in khat addiction and to unravel the role of khat (or its active components) in regulating innate and adaptive immunity against various infectious agents. It would be further interesting to dissect the mechanism by which khat favours the growth of *M*. *smegmatis*.

Substantial work in this domain will help bring mass awareness about the adverse effect of khat addiction among the population and will add potential credence to different anti-khat addiction campaigns runs by government institutes and NGOs alike.

## Supporting Information

S1 File Sheet A in S1 File: Resistin Levels estimated among the study group which comprised of Khat addicted healthy control, Non khat addicted Healthy control, Non Khat addicted TB patient, Khat Addicted TB patients. Sheet B in S1 File: Resistin Levels detected among Non khat addicted Tb patient at first visit (Vo) (Fig 3) Sheet C in S1 File: Resistin Levels detected among Non khat addicted Tb patient at first visit (V2) (Fig 3) Sheet D in S1 File: Resistin Levels detected among Non khat addicted Tb patient at first visit (V4) (Fig 3) Sheet E in S1 File: Resistin Levels detected among Non khat addicted Tb patient at first visit (V6) (Fig 3) Sheet F in S1 File: a) Statistical analysis for Resistin levels estimated between Khat addicted healthy control and Non khat addicted Healthy control. (Fig 1). b) Statistical analysis for Resistin levels estimated between Non khat addicted Tb patients and Khat addicted Tb Patients. (Fig 2). Sheet G in S1 File: Statistical analysis between Resistin levels and bacillary load among among non khat addicted TB patients. (Fig 4). Sheet H in S1 File: Statistical analysis between Resistin levels and bacillary load among among khat addicted TB patients. (Statistical value mention in the results section on page number 10). Sheet I in S1 File: Animal study to determine the effect of different concentration of khat on M. smegmatis. (Table 1).
(XLSX)
